# Reply to Comment on “Improving clinical diagnosis of early-stage cutaneous melanoma based on Raman spectroscopy”

**DOI:** 10.1038/s41416-019-0431-8

**Published:** 2019-03-22

**Authors:** Inês P. Santos, Peter J. Caspers, Remco van Doorn, Senada Koljenović, Gerwin J. Puppels

**Affiliations:** 1 Department of Dermatology, Erasmus MC, University Medical Center Rotterdam, room Ee-1691, P.O.Box 2040, 3000 CA Rotterdam, The Netherlands; 2 Department of Dermatology, Leiden University Medical Center, Leiden, The Netherlands; 3 Department of Pathology, Erasmus MC, University Medical Center Rotterdam, Rotterdam, The Netherlands; 4 RiverD International B.V., Rotterdam, The Netherlands

**Keywords:** Raman spectroscopy, Melanoma

With great interest we have read the letter by De Giorgi et al.^1^ in which they express their disagreement with the conclusions of our study on the potential value of a Raman spectroscopy device in the clinical diagnosis of cutaneous melanoma.^2^

In our paper, we tested the diagnostic accuracy of Raman spectroscopy, expressed as sensitivity, specificity and number needed to treat, in a set of pigmented skin lesions that were deemed suspicious for melanoma by dermatologists. Whereas current clinical diagnosis and dermoscopy rely on recognition of morphological characteristics, Raman spectroscopy provides information about the molecular composition of pigmented skin lesions. Our results indicate that Raman spectroscopy constitutes a valuable diagnostic tool: all melanomas that were analyzed tested positive with Raman spectroscopy (sensitivity 100%), and the estimated number needed to treat was 2.7 (ratio between the number of lesions tested positive by Raman spectroscopy and the total confirmed melanoma).

De Georgi et al. disagree with our conclusions which state that the diagnostic model based on Raman spectroscopy has enabled greater sensitivity and specificity in melanoma diagnosis, detecting all thin melanomas and reducing the number of unnecessary excisions by more than two-fold compared with the current clinical practice.

They object to the fact that pigmented skin lesions were enrolled in the study after a dermatologist performed a clinical assessment and had excised lesions that were clinically suspicious for melanoma, and state that this does not reflect clinical practice.

They furthermore state that such *“*lesion pre-selection frequently includes many melanomas that are easy to diagnose, and which often have an exceedingly high frequency of malignancies within the lesions examined, thus creating an “artificial” diagnostic setting compared to real practice”.

De Giorgi et al. have misinterpreted the objective of our study and our data set. Our results are based on the use of Raman spectroscopy as an add-on to diagnose dermatologist-selected lesions. We do not want to by-pass the dermatologist. The selection of suspicious lesions by a dermatologist is part of the intended clinical practice. This must not be confused with a bias in the case series used.

The sample set mostly consisted of difficult to classify lesions, including melanoma in situ and dysplastic nevi, deemed suspicious for melanoma based on visual inspection by dermatologists specialized in pigmented skin lesions.

This selection of lesions was in line with the objective of our study; namely to investigate the diagnostic use of Raman spectroscopy as an adjunct technique to distinguish between melanoma and unnecessary diagnostic excisions. This is fully in line with the main conclusion drawn from the results regarding the diagnostic accuracy in an independent validation set, and the possible reduction of the number of unnecessary diagnostic excisions if the Raman instrument were used as an add-on to classify lesions considered suspicious by dermatologists.

De Giorgi et al. also criticize the fact that amelanotic lesions were excluded from analysis.

All lesions that were excised by the dermatologist for suspicion for melanoma were subjected to Raman spectroscopy. This included unpigmented lesions suspicious for amelanotic melanoma. After histopathological evaluation, the lesions diagnosed as non-melanocytic were excluded from analysis (basal cell carcinoma, seborrheic wart, lichenoid keratosis, dermatofibroma, haemangioma, scar), because the aim of the study at this stage was to distinguish between melanoma and non-melanoma melanocytic lesions.

De Giorgi et al. furthermore state that only a melanoma left unexcised represents a clinically relevant false negative diagnosis and that in their experience this does not frequently occur, and likely limited to subjects harboring a clinically “featureless” tumor.

We can only refer to the literature. The accuracy and reproducibility of melanocytic skin lesion diagnosis is poor, in particular among general practitioners, as has been demonstrated in several studies.^3–5^

More evidence-based studies are required to provide data about the role of Raman spectroscopy to improve clinical diagnosis of melanoma in different medical settings, including screening of inconspicuous melanocytic skin lesions. With this study we provide evidence that accurate diagnostic results can be obtained by Raman spectroscopy on pigmented skin lesions selected by dermatologists as suspicious for melanoma. We believe that these results represent an important step towards accurate clinical diagnosis of melanoma.
